# Vitamin D metabolites across the menstrual cycle: a systematic review

**DOI:** 10.1186/s12905-019-0721-6

**Published:** 2019-01-28

**Authors:** Anita Subramanian, Alison D. Gernand

**Affiliations:** Department of Nutritional Sciences, The Pennsylvania State University, 110 Chandlee Laboratory, University Park, PA 16802 USA

**Keywords:** Menstrual period, Menstruation, Calcidiol, Calcitriol, Hydroxycholecalciferol, Reproductive hormones

## Abstract

**Background:**

Accurate estimation of vitamin D status is important for health research and can impact prevention and treatment of deficiency in women of reproductive age. We aimed to assess if blood concentrations of 25-hydroxyvitamin D [25(OH)D] or 1,25-dihydroxyvitamin D [1,25(OH)_2_D] change across the menstrual cycle.

**Methods:**

We conducted a systematic search in PubMed, Web of Science, CAB and BIOSIS of literature published until December 2018 which reported concentrations of vitamin D metabolites at two or more identified points among women with regular menstrual cycles.

**Results:**

Ten longitudinal studies met the inclusion criteria; nine studies measured 1,25(OH)_2_D and five studies measured 25(OH)D. Study size ranged from 5 to 47 subjects, with an age range of 18–47 years. One study found a decrease in concentration of 25(OH)D in the periovulatory and luteal phase. Four studies found no changes in concentrations of 25(OH)D. Two studies found a rise in 1,25(OH)_2_D within the follicular phase, including a 128% increase from day 1 to 15 and a 56% increase from day 0 to 12. Two studies found rises in 1,25(OH)_2_D concentrations from the follicular to luteal phase of 13 and 26%. Five studies did not find any changes in concentrations of 1,25(OH)_2_D.

**Conclusions:**

No conclusion can be drawn on the pattern of 1,25(OH)_2_D concentrations across the normal menstrual cycle due to inconsistencies in study findings. Evidence is currently insufficient to assess 25(OH)D concentrations across the cycle. Future studies should aim to measure 1,25(OH)_2_D and 25(OH)D longitudinally, to understand relationships with other hormones and the potential impact on estimates of vitamin D deficiency.

## Background

Vitamin D deficiency has become a problem of public health concern, with increased prevalence among women of reproductive age and higher rates of deficiency in women compared to men [1–4].

 Estimating vitamin D status accurately is important for public health surveillance and research, and can impact the ability to monitor and improve health outcomes across populations. The Institute of Medicine (IOM) defines vitamin D deficiency as having concentrations of 25-hydroxyvitamin D [25(OH)D] < 30 nmol/l (12 ng/ml) and vitamin D insufficiency as 25(OH)D < 50 nmol/l (20 ng/ml) [5]. Over the last decade, the prevalence of vitamin D insufficiency was high among adult women living in Bangladesh (80%), Sri Lanka (59%), Israel (51–60%), and Germany (58%) [2]. Low vitamin D status is also a concern among women of reproductive age living in the US [4, 6, 7] with a quarter of women being classified as vitamin D insufficient [7]. Of concern, the prevalence of vitamin D deficiency was observed to be higher among non-Hispanic black women (41%) compared to non-Hispanic white women (4%) in the US [7].

Vitamin D from both cutaneous production and oral intake gets metabolized by the liver into 25(OH)D, the major circulating metabolite and primary indicator used for measurement of vitamin D status [8–10]. 25(OH)D is further metabolized in the kidneys to 1,25 dihydroxyvitamin D [1,25(OH)_2_D], the biologically active form of vitamin D that acts as a ligand for its nuclear receptor to promote genetic expression [8–10]. Sources of vitamin D include synthesis in skin during sunlight exposure (specifically to ultraviolet B (UVB) radiation), food, and dietary supplements [11–14]. Salmon, mackerel, cod liver oil, shiitake mushrooms and egg yolk are among the few foods naturally containing vitamin D, leaving skin production, vitamin D fortified foods, or supplements as the major source for most of the world [8, 11, 14]. Many factors increase the risk of vitamin D deficiency including poor dietary intake; vegan diet; low availability of fortified foods; religious and cultural practices that reduce sun exposure; darker skin pigmentation; and living in higher latitudes without UVB radiation in the winter [13, 15, 16].

Vitamin D deficiency has been associated with increased risk of diabetes, cardiovascular disease, cognitive decline, certain types of cancer, depression [9, 11], and adverse pregnancy outcomes [17–22]. Poor vitamin D status has also been associated with early menarche, dysmenorrhea, premenstrual syndrome (PMS), and uterine fibroids [23], mechanistically plausible through vitamin D receptors expressed in the ovary, placenta, and uterus [23–26].

The female menstrual cycle is associated with a series of changes occurring in the uterus and ovaries of females resulting in ovulation and the shedding of the uterine lining when conception does not occur [27, 28]. It is driven by the interaction of several hormones produced by the hypothalamus, anterior pituitary, and ovaries, including follicle stimulating hormone (FSH), luteinizing hormone (LH), estrogen, and progesterone [27, 28]. The menstrual cycle begins with menses and is broadly divided into the follicular (pre-ovulation) and luteal phases (post-ovulation) [27, 28].

Concentrations of micronutrients have been reported to change during the menstrual cycle [29, 30]. Zinc has been reported to vary across the menstrual cycle at different time points with the highest concentrations observed at ovulation [29]. Similarly, cyclic changes in copper have been reported with the highest concentrations occurring during menses; and zinc and copper changes might each be due to changing concentrations of estrogen [29]. Biomarkers used to assess iron status such as hemoglobin, transferrin saturation, and ferritin have also been reported to change during the menstrual cycle with the highest concentrations observed during the luteal phase and lowest during menstruation [30]. These changes are thought to result from variations in ovarian hormones and/or plasma volume [30].

While it is known that 1,25(OH)_2_D concentrations increase two- to three-fold during pregnancy [31], and there are some reports of 25(OH)D and 1,25(OH)_2_D across the menstrual cycle, to the authors’ knowledge these biomarkers have not been well chronicled pre-pregnancy. Because concentrations of several nutrition biomarkers change across the menstrual cycle [29, 30, 32], and because vitamin D is a hormone, we hypothesized that vitamin D metabolites may also have cyclic changes along with reproductive hormones. Thus, our objective was to conduct a systematic review of studies that assessed concentrations of 25(OH)D and/or 1,25(OH)_2_D longitudinally to assess if these vitamin D biomarkers change across the normal menstrual cycle.

## Methods

### Eligibility criteria

Original studies which measured concentrations of 25(OH)D and/or 1,25(OH)_2_D in serum or plasma at two or more identified time points per woman were eligible for inclusion in the review. We included studies of normal, healthy women with regular menstrual cycles (26–35 days), and articles written in English. We excluded any review articles or studies which examined women with a specific health issue (e.g. PMS) or among those with irregular cycles or women who were taking any sort of medication. However, we did not exclude studies among women taking oral contraceptives because this is such a common practice (although notably it introduces difficulty in accurately estimating the phases of the menstrual cycle). If a study included a healthy group that met our criteria and any other group outside the criteria, we included data from the healthy group only. Inclusion of studies was not limited by publication date. All laboratory methods for measurement of vitamin D metabolite concentrations were accepted.

### Search strategy

We conducted a systematic search of literature in PubMed, Web of Science, CAB and BIOSIS of literature published until December 2018. The search strategy used several combinations of keywords and MeSH terms. The search strategy used for PubMed was (“menstrual cycle”[TIAB] OR “menstrual cycle”[Mesh]) AND (“vitamin D”[TIAB] OR “vitamin D”[Mesh] OR “25-hydroxyvitamin D”[TIAB] OR “micronutrients”[TIAB] OR “micronutrients”[Mesh] OR “Hydroxycholecalciferols”[TIAB] OR “Hydroxycholecalciferols”[Mesh] OR “calcitriol”[Mesh] OR “calcitriol”[TIAB]). References in identified papers were also scanned for studies that met eligibility criteria. The articles were initially screened on the basis of title and abstract. After excluding irrelevant articles, full text of the remaining studies was screened for eligibility.

### Data collection

Information and variables of interest were extracted from all eligible studies. The main outcomes of interest were concentrations of 25(OH)D and 1,25(OH_2_)D and changes in these vitamin D metabolites across the menstrual cycle. Values were converted to ng/ml for concentration of 25(OH)D and to pg/ml for 1,25(OH)_2_)D if reported otherwise.

This systematic review was conducted according to PRISMA (Preferred Reporting Items for Systematic Reviews and Meta-Analysis) recommendations [33].

## Results

### Study selection

**Fig. 1:**
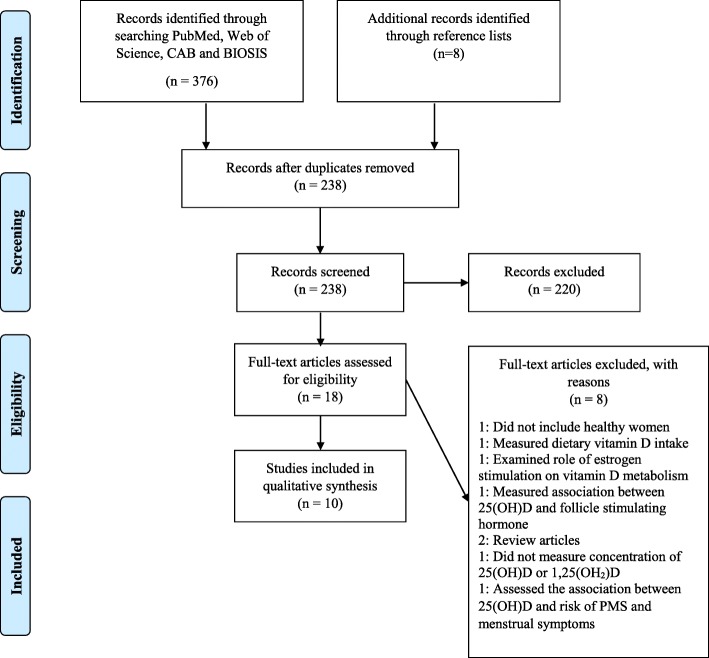
PRISMA Flow Diagram: Flowchart of studies included in the systematic review

We initially identified 376 studies which were retrieved using the search strategies; after removing duplicates, 238 studies remained (Fig. 1). Among these, 220 studies were not relevant on the basis of the title and abstract and were excluded. The remaining 18 studies underwent full review. Eight of these studies did not meet the eligibility criteria and were excluded, leaving ten unique, longitudinal studies included in this systematic review. Five studies measured the concentrations of 1,25(OH)_2_D alone [34–38], one study measured the concentration of 25(OH)D alone [39], and four studies measured the concentrations of both vitamin D metabolites [40–43].

### Study characteristics

Six studies were conducted in the US [34–37, 40, 41], two in Denmark [38, 42], one in Germany [43], and one in Ireland [39]. The study size ranged from 5 to 47 women and the age range across all studies was 18–47 years. The subjects included in the studies were typically a convenience sample of volunteers. Phases of the menstrual cycle were identified by the last menstrual period (LMP) date and/or hormonal evaluation. Eight studies measured vitamin D metabolites among healthy young women with normal menstrual cycles [34–39, 42, 43] and the remaining two studies measured metabolites among both healthy controls and women with PMS [40, 41].

The primary objective of the studies was the following: seven studies aimed to assess the concentrations of vitamin D metabolites and other calciotropic hormones and markers of bone metabolism across the menstrual cycle; one study aimed to assess the association between serum parathyroid hormone (PTH) and changes in endogenous estrogen; one study aimed to assess the effect of estrogens on calcium regulating hormones; and one study aimed to examine the metabolic changes across the menstrual cycle by performing hormonal and nutrient evaluation. All ten studies took at least one measurement within the follicular phase [34–43] and eight studies took measurements that spanned both follicular and luteal phases [35–41, 43].

The summary of findings for the studies are shown in Table 1. Tjellesen et al. [42] reported that the concentrations of 25(OH)D did not change within the follicular phase. Both studies by Thys-Jacobs and colleagues [40, 41] reported no changes in concentrations of 25(OH)D from the early follicular to the late luteal phase. Zittermann et al. [43] reported no changes in 25(OH)D from the early follicular to mid luteal phase. Draper et al. [39] reported a significant decrease in concentration of 25(OH)D in the periovulatory and luteal phase.

**Table 1:** Characteristics of studies which measured vitamin D metabolites longitudinally across the normal menstrual cycle in healthy women^1^

Author (Year)	N	Age range (years)	Method(Sample Type)	Mean (SD)25(OH)D (ng/ml)	Mean (SD)1,25(OH)_2_D (pg/ml)	Main Findings
Baran (1980) [34]	12	19–34	RLRA (Serum)	NA	EF (Day 3): 47 (3)^4^ LF (Day 13): 51 (3)	No change in concentrations of 1,25 (OH)_2_D from day 3 to day 13
Gray (1982) [35]	7	18–35	RIA (Serum)	NA	EF (Day 1): 53.4 (11.8) ^a;4^ EF (Day 8): 56.3 (12.6) ^a^ EL (Day 15): 121 (20.1) ^b^ LL (Day 22): 86.9 (27.2)	Increase in concentration of 1,25(OH)_2_D from day 1 to day 15 Concentration of 1,25(OH)_2_D at midcycle was higher than days 1 and 8
Tjellesen (1983) [42]	5	23–29	PBA^2^ UV-detection^3^ (Serum)	NR	EF^5^: Mean change: 56% from day 2 to day 14	No change in concentration of 25(OH)D from day 2 to day 14 Concentration of 1,25(OH)_2_D increased from day 2 to day 14
Buchanan (1986) [36]	20	24–44	RRA (Serum)	NA	EF: 34 (3)^a^ LF: 39 (3) EL: 43 (3)^b^ LL: 37 (2)	A rise in the concentration of 1,25(OH)_2_D from the EF to EL phase
Muse (1986) [37]	6	26–39	CRA (Serum)	NA	All: 40.1 (1.7)^4;6^	A slight, non-statistically significant change in 1,25(OH)_2_D across the cycle
Nielsen (1990) [38]	8	20–47	RIA (Serum)	NA	EF: 56.5 (13.5) LF: 57.3 (21.9) EL: 57.6 (14.2) LL: 56.9 (10.0)	No change in concentrations of 1,25 (OH)_2_D across the cycle
Thys-Jacobs (1995) [40]	5	28–45	RRA^2^ RBA^3^ (Serum)	EF (Day 2) Midcycle (Days 12,13,14,15) LL (Day 26) All: 45.6 (12.8)^7^ EF: NR Midcycle: 44.8 (11.6) LF: NR	All: 38.0 (8.8)^7^ EF (Day 2): 30.3 (8.4) Midcycle (Days 12,13,14,15): 38.8 (14.6) LL (Day 26): 37.3 (8.8)	No change in concentrations of 25(OH)D across the cycle Statistical significance not reported for concentrations of 1,25(OH)_2_D across the cycle
Zittermann (2000) [43]	10	25^8^	RRA^2^ HPLC^3^ (Serum)	EF: 10.3 (3.1)^c^ LF: 9.3 (2.6) EL: 9.3 (2.4) ML: 8.8 (2.6) EF (next cycle): 8.4 (3.4)^d^	EF: 17.5 (4.1) LF: 19.4 (5.5) EL: 19.6 (5.1) ML: 18.4 (5.3) EF (next cycle): 17.1 (4.9)	No changes in 25(OH)D within the same cycle; Significant decrease in 25(OH)D from EF of one cycle to EF phase of next cycle No change in concentrations of 1,25(OH)_2_D across the cycle
Thys-Jacobs (2007) [41]	47	18–45	RRA^2^ CLA^3^ (Serum)	EF (Days 2 and 7)^9^ Midcycle (Days 12,13,14,15) ML to LL (Days 22 and 27)^10^ All: 26.1 (10.8)^11^	EF (Days 2 and 7) Midcycle (Days 12,13,14,15) ML to LL (Days 22 and 27) All: 49.0 (10.7)^11^ EF: 46.4^a;12^ LL: 52.1^b;12^	No change in concentrations of 25(OH)D across the cycle Significant rise in concentrations of 1,25(OH)_2_D from EF to LL phase
Draper (2018) [39]	34	19–39	CMIA	MP, FP, PO, LP and PP: NR	NA	Significant decrease in concentration of 25(OH)D in the periovulatory and luteal phase

*CRA* cytoreceptor assay, *CLA* chemiluminescence assay, *CMIA* chemiluminescent microparticle immunoassay, *EF* early follicular, *EL* early luteal, *FP* follicular phase, *HPLC* high-performance liquid chromatography, *LF* late follicular, *LL* late luteal, *LP* luteal phase, *ML* mid luteal, *MP*: menstrual phase; *NA* not applicable, *NR* not reported, *PBA* protein binding assay, *PO* periovulatory, *PP* pre-menstrual phase, *RBA* radiobinding assay, *RIA* radioimmunoassay, *RLRA* radioligand receptor assay, *RRA* radioreceptor assay

^1^All studies were conducted in the US except Tjellesen (1983) and Nielsen (1990) conducted in Denmark; Zittermann (2000) conducted in Germany and Draper (2018) conducted in Ireland

^2^Method used to measure concentration of 1,25(OH)_2_D

^3^Method used to measure the concentration of 25(OH)D

^4^Values are presented as mean (SE); SE, Standard Error

^5^Early follicular phase and days with highest concentration of estradiol (number of measurements not reported)

^6^Mean for all measurements across the cycle; measured daily throughout the cycle

^7^Mean for all measurements across the cycle

^8^Mean age reported

^9^Measurement for early follicular phase is approximately between day 6 to 2 days before ovulation

^10^Measurement for late luteal phase is approximately day 27 but varies according to the individual’s cycle

^11^Mean of measurement obtained after determining luteinizing hormone (LH) surge

^12^Standard deviation not reported

^a,b^Concentrations of 1,25(OH)_2_D are significantly different between two time points

^c,d^Concentrations of 25(OH)D are significantly different between two time points

Nine studies measured concentrations of 1,25(OH)_2_D within the follicular phase [34–38, 40–43]. Two studies found a rise in the concentrations of 1,25(OH)_2_D from early in the follicular phase to midcycle [35, 42]. Tjellesen et al. [42] observed a 56% increase in the concentration of 1,25(OH)_2_D from day 2 to day 14, and Gray et al. [35] reported a large 1,25(OH)_2_D increase of 128% from day 1 to 15. Conversely, Baran et al. [34] did not observe a change in concentration of 1,25(OH)_2_D from day 3 to day 13 of the cycle. The other six studies [36–38, 40, 41, 43] did not observe a change in 1,25(OH)_2_D within the follicular phase but however examined 1,25(OH)_2_D across the phases.

Buchanan et al. [36] found a rise of 26% from the early follicular phase to the early luteal phase. Gray et al. [35] reported a large 1,25(OH)_2_D increase of 128% from day 1 to 15 but in the same study a midcycle rise was *not* observed among women taking oral contraceptives. The earlier study by Thys-Jacobs et al. [40] observed a rise of 23% in 1,25(OH)_2_D from the early follicular phase to the late luteal phase, but failed to report statistical significance [40]; their more recent and larger study reported a modest 1,25(OH)_2_D increase of 13% that was statistically significant [41]. Interestingly, Muse et al. [37] measured the concentrations of 1,25(OH)_2_D throughout the menstrual cycle through daily blood collection but only observed a slight, non-statistically significant change across the cycle. The variation in concentrations followed no definite pattern and no midcycle rise was observed [37]. In contrast, Nielsen et al. [38] and Zittermann et al. [43] did not observe any change in concentrations of 1,25(OH)_2_D across the cycle.

## Discussion

After a systematic review of literature on PubMed and other databases, we identified ten longitudinal studies examining concentrations of 25(OH)D and/or 1,25(OH)_2_D across the normal menstrual cycle in healthy women. Five studies measured 25(OH)D, only one study reported a significant decrease in the periovulatory and luteal phase [39] and four studies found no changes in concentrations within a menstrual cycle [40–43]. Four of the nine studies measuring 1,25(OH)_2_D found that concentrations of 1,25(OH)_2_D increased across the menstrual cycle, within the follicular phase [35, 42] and/or from early to late in the cycle [36, 41]. However, five studies did not observe a change in 1,25(OH)_2_D concentrations across any two cycle measurements [34, 37, 38, 40, 43], leaving an inconsistency in findings and no emerging pattern.

Our findings for 25(OH)D are similar to that of another study which examined change in concentrations of 25(OH)D during the follicular phase of the menstrual cycle among patients who were undergoing a modified natural cycle in vitro fertilization (IVF) [44]. The results suggested that concentrations of 25(OH)D, free 25(OH)D, bioavailable 25(OH)D and vitamin D binding protein (DBP) did not significantly change during the early, mid, and late follicular phase of the cycle when the estrogen levels change [44]. Two additional studies examined 25(OH)D across mensuration using a cross-sectional approach but did not test for changes in concentrations of 25(OH)D [45, 46]. Another cross-sectional study examined 25(OH)D in the early follicular phase but did not look at within phase differences [47]. Of note, one study in this review reported a decrease in 25(OH)D from the same point early in one cycle to the next, but it was presumably due to normal seasonal changes (the study was conducted from January to February, when 25(OH)D typically decreases) and was not within a cycle [43]. Only one study in this review reported a significant decrease in concentration of 25(OH)D in the periovulatory and luteal phase [39]. In the same study, concentrations of 25(OH)D were reported to be higher during menses [39]. The decrease in 25(OH)D found in this study, may be due to changes occurring in concentrations of estrogen and progesterone [48].

During the follicular phase, two out of nine studies in this review reported an increase in 1,25(OH)_2_D [35, 42]. We also found, in two studies out of seven that examined both menstrual phases, that concentrations of 1,25(OH)_2_D increased from the follicular phase to the luteal phase [36, 41]. In one of these studies, the concentration of 1,25(OH)_2_D was found to consistently increase from the follicular phase to luteal phase among normal women, but in the same study, a significant decrease was observed in the concentrations of 1,25(OH)_2_D during the early luteal phase among women with premenstrual dysphoric syndrome (PMDD) [41]. Conversely, a significantly higher concentration of 1,25(OH)_2_D was found during the early follicular phase among women with PMS than healthy controls [40]. A possible explanation for this change can also be related to the increased concentration of estrogen during the late follicular phase leading to a rise in 1,25(OH)_2_D [49]. Two of the studies which observed a change in 1,25(OH)_2_D, had a comparatively larger sample size which might have had an impact on the findings [36, 41]. Otherwise there were no apparent connections between studies which did and did not find changes in concentrations of 1,25(OH)_2_D.

Estrogens can increase the activity of 1α-hydroxylase in the kidneys (the enzyme responsible for 25(OH)D to 1,25(OH)_2_D conversion) thereby impacting vitamin D metabolism [49]. Of note, a midcycle 1,25(OH)_2_D rise was not observed among women taking an oral contraceptive (albeit findings from only one study) [35], lending evidence to the hypothesis that changes in estrogens may be needed to drive 1,25(OH)_2_D changes. Additionally, PTH plays an important role in 1,25(OH)_2_D regulation and has a notable midcycle rise [50]. PTH, like estrogen, can also stimulate the production of 1,25(OH)_2_D by increasing the expression of renal 1α-hydroxylase [50]. Overall, more research is needed to understand the determinants of 1,25(OH)_2_D changes, in both normal and pathological female reproductive cycles.

Reproductive hormones change across the menstrual cycle which can have an effect on changes in vitamin D metabolites across the cycle, or vice versa. The antimullerian hormone (AMH) may have an association with 25(OH)D and thereby impact its metabolism across the menstrual cycle [51]. AMH primarily plays a role in inhibiting primordial follicle recruitment which results in a gradual decrease in follicular growth thereby delaying atresia [51, 52]. It has been suggested that vitamin D could be responsible for the regulation of AMH expression, because the AMH gene consists of a domain for the vitamin D response element in the promoter region [51]. This link could explain the apparent impact of vitamin D on ovarian function and menstrual regularity. An association has been observed between serum AMH and serum 25(OH)D among late reproductive aged women suggesting that lower concentrations of 25(OH)D can result in lower ovarian reserve [53]. 25(OH)D has also been found to be related with follicle stimulating hormone (FSH) among premenopausal women [54]. Urinary FSH is considered as a main biomarker of ovarian reserve, and a decrease in concentration of 25(OH)D has been shown to be related with higher FSH levels which can lead to reduction in primordial follicles [54]. This suggests that plasma concentrations of 25(OH)D might have an association with the ovarian reserve and thereby have an effect on women’s fertility [54]. Vitamin D metabolism may also be impacted by concentrations of progesterone [48], which is higher during the luteal phase of the menstrual cycle [27]. An inverse association has been reported between 25(OH)D and progesterone suggesting that lower progesterone is related to higher 25(OH)D during the luteal phase [48]. Further research is needed to investigate mechanisms.

A final factor that we propose could be impacting concentrations of vitamin D metabolites is plasma volume, which has been shown to change across the menstrual cycle [55, 56] as well as impact concentrations of some micronutrients [29, 30, 32]. Therefore, it is currently unknown if changes in concentrations of these plasma-based biomarkers are in part due to plasma volume changes. Overall, there are several plausible mechanisms to suggest that vitamin D metabolite concentrations might be changing across the menstrual cycle and interacting with reproductive hormones, but the exact mechanisms are yet to be elucidated.

There are many limitations to the current body of evidence; first and foremost, few studies met our longitudinal eligibility criteria and most studies had a very small sample size. Reporting was often poor with details not provided on the number of measurements or the specific means and SDs for each measurement. All studies were in high-income countries, limiting generalizability. Very few studies measured the concentration of 25(OH)D, the metabolite for which we were most interested in due to implications for understanding vitamin D status and classifying deficiency. None of the studies used liquid chromatography tandem mass spectrometry, which is considered the most valid method. Seasonal variability can influence vitamin D status which was only reported by few studies. Only one study reported details on intake of supplemental vitamin D.

## Conclusions

We found few studies that have examined vitamin D metabolites across the menstrual cycle, providing limited to insufficient data to understand potential changes or lack thereof. In the existing research, 1,25(OH)_2_D concentrations increased across the menstrual cycle in a few studies, but did not change in others. 25(OH)D concentrations changed across the cycle in one study but not others. Additional studies are needed to better understand 25(OH)D and 1,25(OH)_2_D in menstruating women. Future work should involve longitudinal studies powered to examine both metabolites (at minimum) at least two points across the menstrual cycle using the robust lab methods. Other factors that may be related to concentrations should be investigated, including the woman’s age, race/ethnicity, BMI, and parity. If concentrations of 25(OH)D were found to change across the cycle, this could impact estimates of vitamin D deficiency as well as findings of associations between vitamin D status and health outcomes in women of reproductive age—both important factors in research. Vitamin D deficiency is a worldwide problem, particularly in women during reproductive years, and additional research specific to the menstrual cycle is needed to inform public health recommendations and improve research methods.

## Abbreviations

1,25(OH)_2_D: 1,25-dihyroxyvitamin D
25(OH)D: 25-hydroxyvitamin D
AMH: Antimullerian hormone
FSH: Follicle stimulating hormone
IOM: Institute of Medicine
IVF: In vitro fertilization
LH: Luteinizing hormone
LMP: Last menstrual period
PMDD: Premenstrual dysphoric syndrome
PMS: Premenstrual syndrome
PRISMA: Preferred Reporting Items for Systematic Reviews and Meta-Analysis
PTH: Parathyroid hormone
UVB: Ultraviolet B
